# Arbuscular Mycorrhizal Fungi Mediated Enhanced Biomass, Root Morphological Traits and Nutrient Uptake under Drought Stress: A Meta-Analysis

**DOI:** 10.3390/jof8070660

**Published:** 2022-06-23

**Authors:** Murugesan Chandrasekaran

**Affiliations:** Department of Food Science and Biotechnology, Sejong University, 209-Neundong-ro, Gwangjin-gu, Seoul 05006, Korea; chandrubdubio@sejong.ac.kr

**Keywords:** arbuscular mycorrhizal fungi, biomass, drought, meta-analysis, phosphorous uptake, root morphology

## Abstract

Drought stress remains the major constraint in affecting crop productivity in several arid and semi-arid areas highlighting climate change perspectives. Arbuscular mycorrhizal fungi (AMF) belong to a versatile class of plant–fungal symbiotic associations establishing drought stress alleviation. Nevertheless, the mechanistic mode of sustainable agriculture necessitates rigorous assessment for authentic and reproducible plant growth parameters. Understanding the plant growth promotion, root morphological changes, and nutrient uptake response in AMF-inoculated plants to drought is very important for sustainable agriculture. Therefore, conducted a meta-analysis of published research articles for determining the efficacy of AMF in alleviating drought stress. Overall analysis showed that AM inoculated plants had 49% higher plant growth promotion than the non-mycorrhizal plants under drought stress. Biomass analysis depicted the root dry weight increase by 49%, shoot dry weight increase by 54%, and total dry weight increase by 58% indicating plant biomass traits augmentation. Root morphological traits analysis corresponded to increased root length (37%), root surface (31%), and root volume (65%). Notably, nutrient uptake assessment showed variable increases in uptake patterns such as P uptake by 86%, N uptake by 35%, and K uptake by 46%. Furthermore, the prominent efficacy of AMF was significantly larger under drought for P uptake (*p* < 0.001) and root volume (*p* < 0.001) indicating the linear relationship between root length and P uptake. Thus, the present meta-analysis confirms that drought stress alleviation emancipated by AMF is mediated by root traits modification and phosphorous acquisition efficacy. Hence, meta-analyses along with experimental validations with field trial evaluations will certainly provide the AMF research for escalated applications for better plant productivity, stress alleviation, and sustainable agriculture.

## 1. Introduction

Plant growth is affected by various abiotic stresses such as drought, salinity, temperature, etc., which reduce plant growth promotion and account for the loss in agricultural productivity [1]. Drought stress remains the major constraint for crop productivity in several arid and semi-arid areas [2,3,4]. Further, the drought stress is intensified by the increased human population and reduction in groundwater resources [5]. The adverse effects of drought stress include plant water status, seed germination, morphological traits, biomass variations, soil nutrient acquisitions, photosynthesis, and other physiological mechanisms which subsequently change the normal growth, development, survival, and productivity of the plants [6,7]. Among soil microbes, arbuscular mycorrhizal fungi (AMF) contribute significantly to mitigating the limitations of productive agriculture by drought stress [8,9,10].

AMF are important groups of soil microbes in a symbiotic relationship with plant roots and play a significant role in ecosystems through nutrient cycling. AMF symbiotic interactions have been associated with a wide variety of plants including flowering plants, bryophytes, and ferns utilizing vesicles and arbuscules, the network of hyphae in roots, and typical spores in the rhizosphere [11,12]. Thus, AMF provide a supportive environment in the plants even under unsuitable environments prevailing in the root cells and improves the soil quality, structure, and texture, consequently restoring plant protection and health [13,14,15,16]. The symbiotic association established by AMF is responsible for the sustainability of soil plants across various climatic and environmental conditions [17,18].

Nonetheless, the AMF benefits of plant growth vary considerably among various studies indicating a deeper insight into phenomenal associations that shed light on the AMF interactions with the host plant [19]. The host plant’s unique identity and trait modification of AMF are accounted for effective symbiosis [20]. Several studies regarding drought stress alleviation, nutrient uptake, photosynthetic machinery, antioxidant levels, stomatal conductance, and morphometric parameters have been reported [9,10,13,14,15]. Nevertheless, AMF inoculation has been addressed as a powerful association with stress alleviation [9,21]. On the contrary, reports have stressed the high AMF symbiotic diversity despite root trait variations and drought resistance [22]. Hence research pertaining to AMF and beneficial perspectives have several interesting levels that need to be unraveled.

Meta-analysis associates profuse data analysis and the origin of a quantitative synthesis in summarizing novel and unique consent for the multitude of research viewpoints [23]. The previous meta-analysis based on soil stability and aggregation has largely been implicated for traits modification using AMF for root traits analysis [24]. Another meta-analysis reports involving the efficacy of stress alleviation by AMF symbiosis based on the type of plant, type of soil, type of the AMF inoculum, and the degree of salinity [25]. Earlier, our meta-analytic research has proved traits for enhanced biomass and salinity stress tolerance [26,27]. Jayne and Quigley’s [28] meta-analysis study showed the plant growth and reproductive responses of mycorrhizal colonization to drought stress (54 published articles). However, the present meta-analysis pertains to the random effects assessment of 106 published research articles for determining the efficacy of AMF in alleviating drought stress conditions. Similarly, Augé et al. [29] studied AMF inoculation and alleviation of drought stress but only through stomatal conductance. In the present study, nutritional effects such as phosphorous (P), nitrogen (N), and potassium (K) uptake, biomass and root morphological traits were taken into consideration for meta-analysis. Therefore, this study will give more information on plant growth and nutrient uptake efficiency of AMF under drought stress. Moreover, studies addressing the effects of AMF inoculation on different drought stress levels and their traits assessment involving plant biomass, nutrient uptake, and root morphological traits are scarce. Our meta-analysis was planned to intrigue the below listed key questions: (1) What is the status of AMF symbiosis in promoting plant biomass, nutrient uptake, and root morphology across various studies? (2) How the AM effect varies and proves distinct during different levels of drought? (3) What is the relationship between P uptake and root traits? (4) How do plant and fungal traits affect plant growth under drought stress? Thus, the key questions were designed to correlate the multiple factors for the effectiveness of AMF symbiosis and drought tolerance.

## 2. Materials and Methods

### 2.1. Literature Search and Eligibility Criteria

Research publications were from the Web of Science, Google, Google Scholar, Science Direct, Springer, Taylor and Francis, Wiley, Springer, Nature, and Science. The following keywords were used for searching literature: arbuscular mycorrhizal fungi and drought stress or AM fungi* or mycorrhiza* or mycorrhizal inoculation* or AMF inoculation* and drought stress or water stress* and biomass*, nutrient uptake, and drought stress/alleviation/tolerance or stress mitigation*. The use of the Boolean truncation (‘*’) character ensured that the variations of the word such as mycorrhizae, mycorrhizas, mycorrhizal, and so on were included. WebPlotDigitizer v. 4.5 was used to extract data graphs [30]. Percentage of field capacity (FC) was used for classification of the type of drought stress. The FC at 60–80% range is considered mild drought stress, FC at 40–60% range is considered moderate drought stress, and FC at 0–40% is considered severe drought stress. Further, nine fixed response variables were used for analysis comprising morphometric assessment of root, shoot, and total dry weight, root volume, length, and surface area together with nitrogen (N), P, and K uptake (data from both nutrient concentration and nutrient content taken into consideration for nutrient uptake). 

### 2.2. Meta-Analysis

Meta-analysis was performed using MetaWin v2.1 software [31] and the LRR representing the natural log values of response ratio correlates to the mean of the treatment representing AMF inoculation compared to the control (without AMF inoculation). Effect size evaluation and variance calculation were performed using the random-effects model in the meta-analysis. An effect size/response ratio (lnR, the natural log of the ratio of the mean value of a variable of interest in the AMF inoculation to that non-inoculated plants during drought stress) was used to represent the magnitude of the effects of AMF inoculation as follows: lnR = ln (Xi/Xc) = lnXi − lnXc, where Xi and Xc are the response values of each observation in the treatment (with AMF) and control (without AMF), respectively. The corresponding sampling variance for each lnR was calculated to the following equation; vlnR = (1/Ni) × (Si/Xi) + (1/Nc) × (Sc/Xc), where Ni, Nc, Si, and Sc are the sample sizes, standard deviations in the experimental and control groups, respectively.

The analysis utilized 3999 iterations with bootstrapping with 95% confidence intervals (BS CIs) used to estimate the homogeneity variance statistic Q factor corresponding to no-categorical analyses. Chi-square distribution at the *p* < 0.05 level of significance was rendered heterogeneous for further assessments. For each categorical analysis, the total heterogeneity was calculated among studies (Q_T_), within-group heterogeneity (Q_W_), or between-group heterogeneity (Q_B_). Studies were considered significant when Q_B_ was significant (*p* < 0.05) (Q_B_/Q_T_ ≥ 0.1) [31]. Zero (0) effect sizes signify no difference in effects between the experimental and control groups, negative values represent effects where the control group attains a greater significance than the experimental group, and positive values represent effects where the experimental group attains a greater significance than the control group. AMF inoculation effects were estimated as a percentage change, relative to the control (%), using the equation [exp (LRR) − 1] × 100, and *p* < 0.05 values are statistically significant. 

## 3. Results

### 3.1. Overall AM Fungi Inoculation Effects on Alleviation of Drought Stress

**Figure 1:**
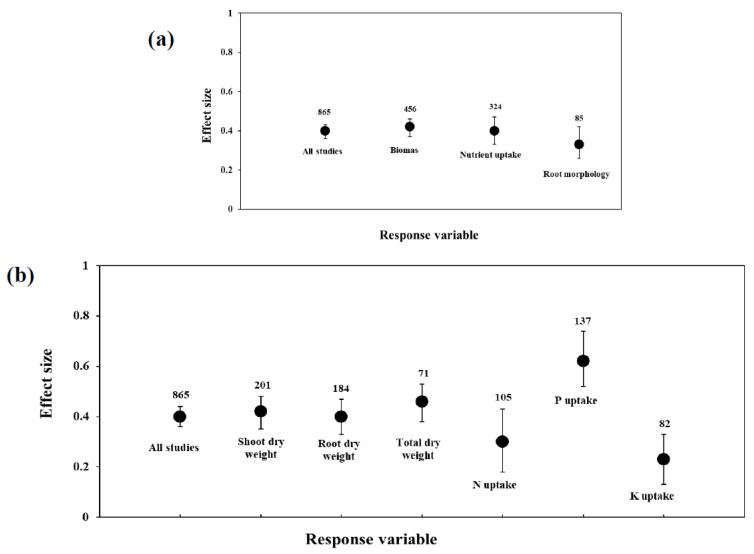
Effect of arbuscular mycorrhizal fungi inoculation responses under drought stress. (**a**) The overall analysis of the dataset. (**b**) Response variables analysis. Error bars are means ± 95% bootstrap confidence intervals (BS CIs). Where the BS CIs do not overlap the horizontal dashed lines, the effect size for a parameter is significant at *p* < 0.05. All response ratios differed significantly from zero. The numbers of trials are shown above the bar.

Our meta-analysis revealed that AMF-inoculated plants illustrate a significant increase in plant growth by 49% across all crop plants under drought stress conditions Figure 1a (Table 1). Furthermore, inoculation with AM fungi increased the shoot, root, and total dry weight by 52%, 49%, and 58%, respectively.

Furthermore, the mean nutrient uptake of AMF-inoculated plants significantly increased by 49% (*p* < 0.01). For, P, N, and K uptake, mean effect size increased by 86%, 35%, and 26%, respectively, determining the effectiveness of AMF inoculation and nutrient uptake under drought stress (Figure 1b). Moreover, significant positive effects on root lengths, root volume, and root surface area increased by 37%, 65%, and 31%, respectively. The level of drought stress significantly modified the AMF inoculation efficiency on plant growth in crop plants. The overall analysis of the level of drought stress showed that severe levels of drought stress (45%) had less increase in plant growth in crop plants than moderate (51%) levels of drought stress and mild level of stress (54%) showed the highest mean effect in crop plants.

### 3.2. Influence of Stress Level on Biomass and Nutrient Uptake

**Figure 2:**
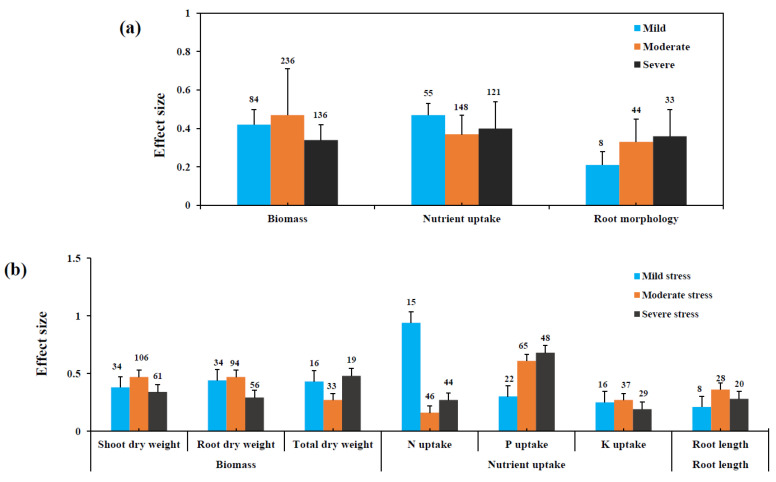
Effect of levels of drought stress on response variables. (**a**) Levels of stress on biomass, nutrient uptake, and root morphology. (**b**) Levels of stress on different response variables.

For plant biomass, moderate levels of stress (60%) showed the highest mean effect size than those of mild (52%) and severe (40%) levels of stress (Figure 2a) in AMF-inoculated plants. Among plant biomass, shoot dry weight showed the highest mean effect size at moderate (60%) levels of stress than those of mild (46%) and severe (40%) levels of stress. Root dry weight showed a similar kind of variation among studies in biomass variations according to stress patterns. In contrast, total dry weight showed the highest mean effect size at severe (61%) levels of stress followed by mild (54%) and moderate (31%) levels of stress (Figure 2b). For root traits, AMF-inoculated plants at a moderate level of drought stress showed that the mean effect size of root length increased by 43% and at severe and mild levels of stress by 32% and 23%, respectively, in root length augmentation. 

Subgroup meta-analyses of the nutrient uptake also showed a considerable variation in the level of stress (Figure 2a). The mild level of drought stress (60%) showed the highest level of nutrient uptake followed by the severe level of stress (49%), while the moderate level of stress yielded the lowest concentration of nutrient uptake (45%). Regarding the P uptake in host plants, a severe level of drought stress showed the highest mean effect size (97%) than those of moderate and mild levels of stress by 84% and 35%, respectively. 

**Figure 3:**
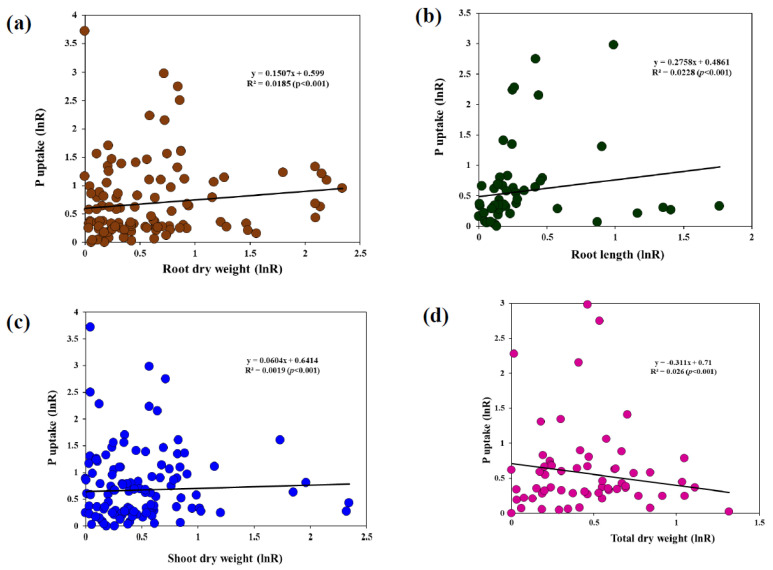
Correlation analysis. (**a**) P uptake vs. root dry weight; (**b**) P uptake vs. root length; (**c**) P uptake vs. shoot dry weight; (**d**) P uptake vs. total dry weight.

Regarding N uptake, mild stress showed the highest level of mean effect size, and due to the low sample size, the analysis was performed with more precautions to avoid errors. Moreover, K uptake showed similar results in both mild (28%) and moderate (31%) levels of stress, the least effect was observed at severe (21%) level of drought stress (Figure 2b). In addition, root length showed a considerable variation in AMF inoculation efficiency, in which moderate level of stress highest level of effect size followed by severe and mild stress (Figure 2b). In addition, a linear correlation between the root length and P uptake was found in the present study. A significant linear correlation was also observed between P uptake and shoot, root, and total dry weight of AMF-inoculated plants under drought (Figure 3).

### 3.3. Influence of Drought Stress Level on AMF and Plant Variables

**Figure 4:**
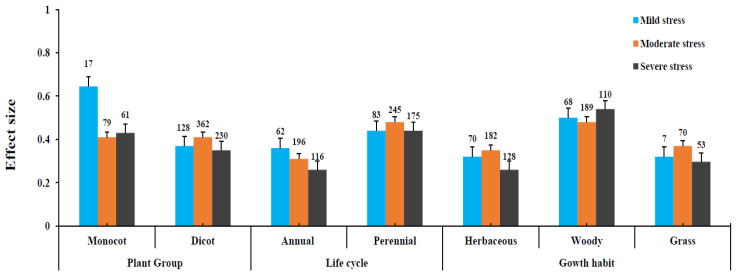
Categorical analysis of plant group, life cycle, and growth habit under different levels of stress.

The levels of stress were verified to the mean effects of plant subgroups differed considerably. No significant mean effect for monocot vs. dicot in the moderate and severe levels of stress was found and mild levels of stress showed a significant positive effect on plant growth, particularly for monocot plants. However, among life cycle, perennial plants had more positive effects than those annual plants. The mean effect increased by 62% for moderator levels of stress but both low and severe levels of stress yielded similar results (55%). Moreover, categorical analysis of growth habits such as herbaceous, woody, and grass plants showed a significant positive effect on all three levels of stress. Among growth habits, woody plants showed the highest level of inoculation response in all three levels of stress: 65%, 62%, and 72%, respectively (Figure 4). 

**Figure 5:**
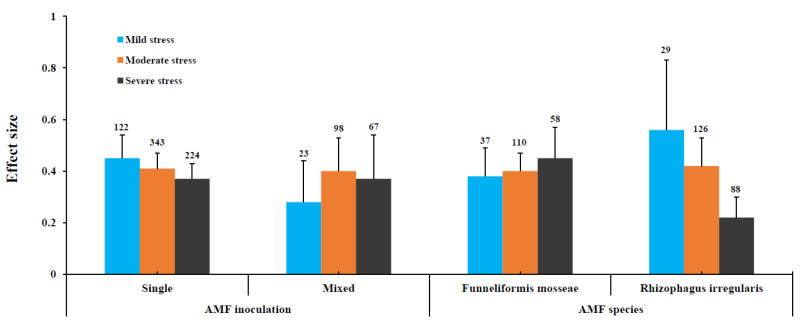
Categorical analysis of AMF inoculation and AMF species under different levels of stress.

Herbaceous plants revealed a moderate level of stress found to be efficient compared to those of mild and severe levels of drought for AM effect. A similar kind of result was observed for grass plants under three different levels of drought stress. Among AMF species, the two most studied AMF species, *Rhizophagus irregularis* and *Funneliformis mosseae*, were used for further analysis (Figure 5). Our results showed a reduced trend in the effect size for *R. irregularis* at a mild level of drought stress, which showed the highest effect size and lowest effect size found for severe levels of drought stress. Whereas, the opposite trend was found for *F. mosseae*, in which severe levels of drought stress showed the highest effect size and mild levels of stress showed the lowest effect size. Among AMF inoculation, single species inoculation showed a reduced trend depending on the level of drought stress, whereas mixed-species inoculation showed the highest effect size at a moderate level of drought stress followed by the severe and low level of drought stress. It suggests the variation among AMF species and between AMF species at different levels of drought stress.

## 4. Discussion

AMF colonization depends on the nutrient availability, host specificity, plant genotypes, and changes in nutrient uptake under different environmental conditions revealing the significance of AMF–plant host interactions [32,33]. However, symbiotic associations between AMF–hosts are prominent during stress situations and unavailability of nutritional consistency [34,35]. AMF efficiency on ideal plant growth, diverse nutrient uptake, and water acquisition ability indicate positive outcomes [36,37]. Nonetheless, the commanding benefits in plant growth vary considerably among various studies indicating a deeper insight into phenomenal associations between the host plant and AMF, depending on the host plant’s unique identity and trait modification of AMF [19,20]. On the contrary, reports have stressed the high AMF symbiotic diversity, in spite of root trait variations, and drought resistance [22].

### 4.1. Overall Efficiency of AMF on Plant Growth under Drought Stress Conditions

Natural calamities and global warming have expected the clear need for food safety, security, and plant productivity for sustaining the global population and crop production. Global warming accounts for drought stress conditions and the necessity for drought stress management for better food production and supply needs. Hence, sustainable practices for managing food production have been stressed by food technologists and scientists, globally [38]. AMF belong to the most utilized symbiotic association for plant growth promotion and drought resistance [39,40,41]. The present meta-analysis affirms that AMF inoculation ameliorates drought stress and promotes potential plant growth under stressed conditions. AMF enhanced plant growth and the accumulation of shoot and root biomasses, which is consistent with the results of previous studies showing that AMF can alleviate the negative effects of drought and improve plant growth [39,40,41,42,43,44]. Yang et al. (2014) [42] showed that AM fungi inoculation significantly increased the plant biomass at varying stress levels. Furthermore, Quiroga et al. [43] confirmed that AMF colonization influences root dry weight in all drought stress levels, compared to non-inoculated plants. Thus, it will be helpful for extra-radical hyphae persistence and increased surface area of the mycelia network of the mycorrhiza for root biomass changes [38,42,44,45]. 

Among plant species, *L. sativa* showed the highest effects for nutrient uptake across all studies, indicating an increase in the shoot biomass and P uptake. Whereas, other plant species such as *P. trifoliate*, *Z. mays*, *C. annum, S. lycopersicum*, and *G. max* showed significant effect but less pronounced. Thus, the study emphasizes inter-species and intra-species variations, reproducible field trials for the authenticity of AMF inoculation, and precise assessment for biomass enumeration. The critical question to be discussed is based on AM effects variations among plant parts and their potential implications. The results show positive effects on life cycle (*p* = 0.004) and growth habits (*p* = 0.001), significantly. Perennial plants and woody plants had the highest effect on plant growth promotion compared to other plant functional groups under different levels of drought stress. AMF inoculation increases the nutrient availability and growth promotion in woody tree seedlings corresponding to plant height and their basal diameter, enhanced biomass accumulation, and resistance to stress mechanisms for root expansions of fibrous roots [10,38]. 

AM effects in inoculated crop plants may significantly vary depending on the identity of AM fungi. *F. mosseae*, *D. versiformis*, and *R. irregularis* showed variations in positive outcomes. The mycorrhizal contribution of *F. mosseae* increased with the increase in drought stress, whereas *R. irregularis* contribution was found to be stable. Moreover, *F. mosseae* showed the highest effect size for biomass, whereas *R. irregularis* showed the highest effect size for the nutrient uptake. Moreover, biomass and root morphology with mixed inoculation of AMF species had the highest effect compared to single inoculation. Nutrient uptake had the highest efficiency in the single inoculation than mixed inoculation. The characteristic inoculation and the corresponding effect can be attributed to the ecotype with broad-spectrum ecological adaptations [46]. Hence, *F. mosseae* and *R. irregularis* can have potential ecological implications based on the environmental status. The significant reduction in drought stress in crop plants can be explained by increased biomass resulting from AM improved growth due to enhanced nutrient supply.

### 4.2. Nutrient Uptake and Plant Growth Responses

Drought stress delays minerals and nutrient uptake and accessibility for the root surfaces due to dry areas around roots and waterless conditions. Hence, AMF can positively regulate nutrient uptake to a greater extent [12,47]. AMF inoculation significantly increased the nutrient uptake under different levels of drought stress conditions. Among the three nutrient uptakes analyzed, the mycorrhizal contributions to the uptake of P were higher than the uptake of N and K. AMF-inoculated plants were proved for escalated plant growth by an increase in N, P, and K uptake during drought stress [38,48,49,50]. Nitrogen and phosphorous are the principal nutrients of plant cells that render the cell characters by altering cell structure and functions for growth patterns [42,48,51]. AMF colonization enhanced P concentration under all treatments. Importantly, our analysis proved that the relative impact of AMF on plant P nutrition was larger in AMF-inoculated plants, likely due to an overall lower nutrient mobility under drought conditions. Moreover, the substantially higher P uptake across all drought stress levels shows that AM fungi are effective at increasing plant P interception in a wide range of soil moisture conditions. The effect of AM fungi on P uptake was especially increased in the severe drought stress level, in which soil moisture was more variable and had greater extremes of dryness. According to Bunemann et al. [52], the larger extent of drought may cause outbursts of P availability that could be well exploited by AM fungi than roots alone. AMF colonization enhanced N concentrations under all treatments, which likely contributed to the higher photosynthetic rates under drought conditions. N is an essential constituent of proteins in plant tissues and Rubisco, the key enzyme of the Calvin–Benson cycle, which represents almost 50% of total leaf proteins, and the increased K^+^ levels effectuate the up-regulation of plant defense, osmolyte accumulation, antioxidant profiles, and enzymatic regulation of plant metabolism [33,53]. Ruiz-Lozano and Azc’on [54] reported that the protection of AM plants against water stress is due to partial K uptake related to stomatal movement. In addition, El-Mesbahi et al. [55] showed that increased uptake of K in AMF-inoculated plants could be a mechanism for water transport by mycorrhizal hyphae because increased K uptake enhanced the root hydraulic conductivity more in AM plants than non-mycorrhizal plants, regardless of the drought stress conditions. However, the activity of the significant effects varies between studies and depends on the identity and traits of the plant–fungal partners involved in AMF symbiosis [20,56]. 

The contribution of AMF inoculation to nutrient uptake by plants varied much depending on fungal species, plant species, and level of stress status. The contribution of *R. irregularis* to nutrient uptake was the highest, followed by *D. versiformis* and *F. mosseae*. Hao et al. [57] also proved that *R. irregularis* inoculation significantly increased plant shoot and root P concentration and contents. Among plant species, *L. sativa* showed the highest mean effect, followed by *Z. mays* and *S. lycopersicum* under drought stress conditions. Significant positive effects on plant species for P uptake under drought stress conditions were observed. Studies reported that mycorrhizal symbiosis positively increased the concentrations of nutrients in several species under drought stress [9,10,12,39,47,54,56]. Mycorrhizal contribution to nutrient uptake was higher in AMF-inoculated plants than in non-mycorrhizal plants under drought stress, explaining the better growth performance and biomass accumulation of mycorrhizal plants under drought stress. Thus, the meta-analysis strongly suggests that the increased drought tolerance of plants can be attributed to the improved nutrient uptake. The positive relationship between mycorrhizal contribution to the plant biomass and mycorrhizal contribution to nutrient uptake is affirmative. Hence, the improved nutrient uptake contributes to the increased drought tolerance of plants as evident from the meta-analysis.

### 4.3. Relationship between Root Morphology and P Uptake

Morphological and physiological changes in plants under drought stress conditions were accounted for improved root traits. AMF have been proven for plant biomass or production, but studies focusing on roots are scarce about drought stress alleviation [10,58,59]. AMF promote plant growth by increasing root growth thereby augmenting biomass and mineral uptake in combating stress environments [38]. The root dry weight increased in the AMF treatment under different levels of drought stress. Low water availability can be corroborated by the reduction in primary root elongation and subsequently water uptake [60]. Plant roots than shoots are accounted more for mitigation. The root growth of plants was significantly enhanced particularly in *F. mosseae* followed by *R. irregularis* inoculated plants (*p* = 0.03). Al-Karaki and Al-Raddad [61] reported that the root dry weight and total root length of wheat genotypes were reduced by drought stress and *F. mosseae* had a higher root dry weight than non-mycorrhizal plants, proving the similar results that roots are more augmented than shoots. According to Augé [13], AMF inoculation under drought increased the root hydraulic conductivity, which is helpful for the effective absorption of soil water and transport to the plant. The enhanced root absorptions result from the larger root system due to AMF hyphae, which can increase the area beyond the root zone, thus increasing the available volume of the soil solution [29,62,63]. Augé [13] and Zhu et al. [64] also showed that increased levels of relative water content in AMF-inoculated plants may be vital for moving water from root to leaf surfaces and stomatal opening which is useful for dehydration avoidance under drought stress. Thus, mycorrhizal root development and distribution in soils are important for root-water and nutrient uptake studies in soil–plant systems emphasizing the great root surface area and volume than non-AMF-inoculated plants under drought stress conditions. The improved effect of AMF on the root morphology was considerably greater under drought stress, indicating the greater importance of mycorrhizas in root modification under drought stress conditions [65,66,67].

## 5. Conclusions

Our meta-analysis results proved that AMF inoculation significantly increased the plant biomass, nutrient uptake particularly P uptake, and root morphological characteristics under different levels of drought stress. Moreover, root dry weight and root length had a significant linear relationship with P uptake under different levels of drought stress. Thus, the present meta-analysis reveals the P uptake efficiency as a direct mechanism and root modifications as the indirect mechanism in AMF symbiosis for drought stress alleviation. However, further studies on plant and AM fungi species variations, types of inoculants, different levels of drought intensities on plant growth, and drought stress resistance mechanisms with appropriate field trials and experimental evidence are necessitated. Hence, meta-analyses along with field trial evaluations will certainly be helpful for AMF-related studies for better agricultural productivity, stress alleviation, and sustainable agriculture.

## Figures and Tables

**Table 1:** AMF inoculation effect on plant growth and nutrient uptake under drought stress.

Analysis	Response Variable	df	Effect Size	Q_total_	Prob _(Chi-Square)_
Overall studies	Overall studies	864	0.4026	517.474	1.0000
Biomass	Shoot dry weight	200	0.4261	274.76	0.0003
	Root dry weight	183	0.3968	273.58	0.0001
	Total dry weight	70	0.4428	81.59	0.1
Nutrient uptake	N uptake	104	0.3003	231.5	0.0001
	P uptake	136	0.6239	219.79	0.0001
	K uptake	81	0.2270	24.057	1.0000
Root characteristics	Root length	55	0.3155	108.9	0.00002
	Root volume	13	0.5031	12.65	0.475
	Root surface area	14	0.2748	40.64	0.0002

## Data Availability

The data are available from corresponding author, upon resonable request.
